# Meta‐analysis of cryoballoon ablation versus antiarrhythmic drugs as initial therapy for symptomatic atrial fibrillation

**DOI:** 10.1002/clc.23695

**Published:** 2021-07-29

**Authors:** Yin‐jun Mao, Wei‐ye Feng, Qun‐ying Huang, Fu‐ling Yu, Jian‐xing Chen, Hang Wang

**Affiliations:** ^1^ Department of Pharmacy First Affiliated Hospital of Fujian Medical University Fuzhou China; ^2^ Department of Cardiology First Affiliated Hospital of Fujian Medical University Fuzhou China; ^3^ Department of Anesthesiology First Affiliated Hospital of Fujian Medical University Fuzhou China

**Keywords:** antiarrhythmic drugs, atrial fibrillation, cryoballoon ablation, initial therapy

## Abstract

**Background:**

The optimal first‐line approach for patients with symptomatic atrial fibrillation (AF) remains unclear. We compared the efficacy and safety of cryoballoon ablation (CBA) and antiarrhythmic drugs (AADs) in the initial management of symptomatic AF.

**Hypothesis:**

CBA is superior to AAD as initial therapy for symptomatic AF.

**Methods:**

We searched the EMBASE, PubMed, and Cochrane Library databases for randomized controlled trials (RCTs) that compared CBA with AAD as first‐line treatment for AF from the date of database establishment until March 18, 2021. The risk ratio (RR) with a 95% confidence interval (CI) was used as a measure of treatment effect.

**Results:**

Three RCTs that enrolled 724 patients in total were included in this meta‐analysis. Majority of the patients were relatively young and had paroxysmal AF. CBA was associated with a significant reduction in the recurrence of atrial arrhythmia compared with AAD therapy, with low heterogeneity (RR, 0.59; 95% CI, 0.49–0.71; *p* < .00001; *I* ^*2*^ = 0%). There was a significant difference in the rate of symptomatic atrial arrhythmia recurrence (RR, 0.44; 95% CI, 0.29–0.65; *p* < .0001; *I* ^*2*^ = 0%); however, the rate of serious adverse events was similar between the two treatment groups (RR: 1.18; 95% CI: 0.71–1.97, *p* = .53; *I* ^2^ = 0%). Transient phrenic nerve palsy occurred in four patients after the CBA procedure.

**Conclusion:**

The current meta‐analysis suggests that CBA is more effective than AAD as initial therapy in patients with symptomatic paroxysmal AF. Serious iatrogenic adverse events are uncommon in CBAs.

## 1 INTRODUCTION

Atrial fibrillation (AF) is among the most common arrhythmias, occurring in approximately 1%–2% of the total population.^1^ It recurs in 90% of treated AF patients without preventive therapy.^2^ The current guidelines recommend the use of antiarrhythmic drugs (AADs) as initial therapy for rhythm control in patients with symptomatic AF.^3^, ^4^ However, AADs have substantial side effects^5^ and a limited efficacy.^6^

Compared with AAD, isolating pulmonary veins through catheter ablation (CA) is superior in maintaining sinus rhythm, preventing AF recurrence, and enhancing the quality of life in patients who have failed drugs.^7^, ^8^, ^9^ Thus, CA has become a recognized strategy for long‐term rhythm control in this population.^10^ In the Early Treatment of AF for Stroke Prevention (EAST‐AFNET 4) trial, cardiovascular outcomes were better with early rhythm control through CA than with conventional treatment.^11^ Ablation for rhythm control may also slow disease progression to persistent AF, which is harder to manage.^12^ Similarly, recent studies have shown that CA intervention prior to pharmacotherapeutic failure is beneficial due to a shorter interval between diagnosis and ablation, resulting in lower incidences of recurrent arrhythmia, hospitalizations, and repeat procedures.^13^, ^14^, ^15^

The two most frequently used ablation techniques, radiofrequency ablation (RFA) and cryoballoon ablation (CBA), differ in their application methods and energy sources. RFA applies radiofrequency currents in a point‐by‐point mode, whereas CBA uses low‐temperature energy on the balloon in a one‐step mode, causing cellular necrosis by tissue heating and freezing, respectively. A previous meta‐analysis of three randomized controlled trials (RCTs) demonstrated that RFA was more effective than AAD as an initial treatment strategy for paroxysmal AF.^16^ However, the aforementioned study reported differences in outcome between RFA and AAD. Whether or not these results can be extrapolated in comparisons of CBA and AAD is uncertain. To address this research gap, we performed a meta‐analysis of RCTs to identify differences in efficacy and safety between CBA and AAD as initial therapy for symptomatic AF.

## 2 METHODS

No ethical approval and patient consent were required.

### 2.1 Database search

This meta‐analysis was conducted based on the preferred reporting items for systematic reviews and meta‐analyses (PRISMA) guidelines.^17^ The EMBASE, PubMed, and Cochrane Library databases were systematically searched for pertinent studies published before March 18, 2021, using the following search terms: “antiarrhythmic drugs,” “randomized,” “cryoballoon ablation,” “catheter ablation,” “initial therapy,” “first‐line,” and “atrial fibrillation.” In addition, we searched for recent major cardiovascular meetings and ClinicalTrials.gov for further potential information. There were no restrictions on publication year or language.

### 2.2 Selection criteria

Studies that met the following criteria were included: (a) RCTs that evaluated CBA versus AAD as initial therapy for symptomatic AF and (b) studies that reported efficacy and/or safety outcomes. Observational studies, reviews, editorials, and letters were excluded. The Cochrane bias risk assessment tool was used to evaluate bias in the included trials.

### 2.3 Data extraction

Two authors (M.‐Y.J. and F.‐W.Y.) independently extracted relevant data from the included studies and reached an agreement on all items. Disagreements were resolved through discussion. The following data were recorded from each trial: study name, year of publication, sample size per group, mean age of patients, proportion of males per group, number of patients who crossed over from the AAD group to the CBA group or vice versa, and duration of follow‐up. Additionally, underlying diseases and cardiac function parameters of subjects were recorded, including hypertension, diabetes mellitus, coronary artery disease, previous thromboembolic events (e.g., transient ischemic attack [TIA], stroke, deep vein thrombosis, and pulmonary embolism), left atrial diameter, and left ventricular ejection fraction.

### 2.4 Study outcomes

The primary outcomes of this study were recurrences of atrial arrhythmia and symptomatic atrial arrhythmia. The secondary outcomes were serious adverse events (SAEs), additional ablation after initial treatment failure with either CBA or AAD, percentage of crossover to the alternative treatment, thromboembolic events, bradycardia, syncope, chest pain, vascular access site hemorrhage, and pericardial complications (e.g., hemorrhage, perforation, effusion, tamponade, pericarditis, and any combinations).

### 2.5 Statistical analysis

The risk ratio (RR) and 95% confidence interval (CI) were calculated to compare the effects of the combined therapy. The Cochrane *I* ^*2*^ statistic was used to analyze the heterogeneity, which was divided into high (*I* ^*2*^ ≥ 75%), moderate (*I* ^*2*^ > 25% and <75%), and low (*I* ^*2*^ ≤ 25%).^18^ The fixed‐effect model was used when the heterogeneity was low. Otherwise, we used a random‐effect model. The Grading of Recommendations Assessment, Development and Evaluation (GRADE)^19^ approach was used to evaluate the quality of evidence for outcomes. Statistical significance was set at *p* < .05. RevMan software (version 5.3.5) was used for all analyses.

## 3 RESULTS

### 3.1 Search results and study characteristics

**FIGURE 1:**
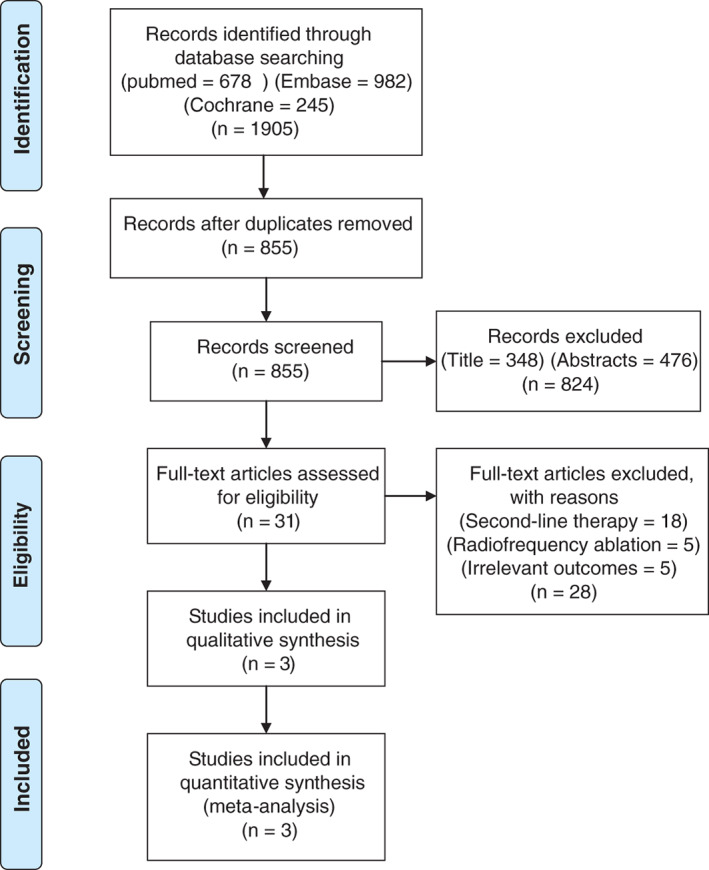
PRISMA flow chart illustrating study selection process

The PRISMA flowchart systematically describes the literature selection process (Figure 1). First, an initial search using a pre‐specified keyword identified 1905 records and deleted 1050 duplicates. Second, 824 records based on title/abstract and 28 records filtered for detailed articles were excluded. Finally, the meta‐analysis included three RCTs published between 2020 and 2021.

The three trials were multicenter and included 724 patients, with 365 in the CBA group and 359 in the AAD group.^20^, ^21^, ^22^ The subjects included in the two of these three trials were limited to Canada^21^ and the United States,^20^ respectively. The remaining Cryo‐FIRST trial expanded its research to other regions, including Australia, Europe, and South America.^22^ Most study participants had paroxysmal AF with mild or no cardiovascular disease. The primary inclusion criterion was a symptomatic AF that was not previously treated with Class I or III AADs. The data related to the underlying disease or cardiac indicators in patients with AF were comparable in both groups. All ablation procedures used a 23‐ or 28‐mm second‐generation cryoballoon (Arctic Front Advance Cardiac Cryoablation Catheter, Medtronic) for pulmonary vein isolation. Among the included studies, the most frequently used AADs were propafenone, flecainide, sotalol, and dofetilide. Amiodarone was excluded due to potential extra‐cardiac toxicity. The blanking period after initial therapy with CBA was 3 months. Except for the Cryo‐FIRST trial,^22^ re‐ablation during the blanking period was considered an endpoint event. The number of patients who crossed over from the AAD group to the CBA group was 89. All three trials were followed up for 1 year. The baseline characteristics of subjects in the included trials are shown in Tables 1 and 2.

**TABLE 1:** Baseline characteristics of the included trials

Study	Year	Patients (*N*)	Male (%)	Mean age (years)	HTN (*N*)	DM (*N*)	LVEF (%)	LAD (mm)	CAD (*N*)	PTE (*N*)	AB in CBA group (*N*)	AB in AAD group (*N*)^a^	Crossover to AAD (*N*)	Crossover to CBA (*N*)	Follow up (months)
STOP AF First	2020	104/99	61/58	60 ± 11/61 ± 11	58/57	15/17	61 ± 6/61 ± 9	39 ± 6/38 ± 5	13/12	2/3	0	34	2	34	12
EARLY‐AF	2020	154/149	73/69	58 ± 12/60 ± 11	57/55	NR	60 ± 7/60 ± 8	40 ± 5/38 ± 7	12/7	4/5	18	19	26	19	12
Cryo‐FIRST	2021	107/111	71/65	51 ± 13/54 ± 13	31/36	1/4	63 ± 5/64 ± 5	37 ± 6/38 ± 5	2/1	0/1	6	36	1	36	12

Abbreviations: AAD, antiarrhythmic drug; AB, additional ablation; CAD, coronary artery disease; CBA, cryoballoon ablation; DM, diabetes mellitus; HTN, hypertension; LAD, left atrial diameter; LVEF, left ventricular ejection fraction; PTE, previous thromboembolic events.

a The number of patients who received additional ablation therapy after the initial AAD therapy failed, that is the number of patients who crossed over to the CBA therapy in the AAD group. Data are presented as patients receiving catheter ablation/patients receiving antiarrhythmic drugs.

**TABLE 2:** Characteristics of eligible studies

Study	Region	Inclusion criteria	Exclusion criteria	CBA technology	AAD therapy	Primary endpoints	Secondary endpoints
STOP AF First	United States	Symptomatic PAF for at least 6 months	Age <18 years or > 80 years, previous treatment with an AAD (Class I or III) for seven or more days, an enlarged LAD (>5 cm), or a previous LA ablation /LA surgery	A second‐generation cryoballoon (Arctic Front Advance Cardiac Cryoablation Catheter, Medtronic)	Flecainide, propafenone, sotalol, dronedarone	Any AA recurrence (documented AF, AT, or AFL for ≥30 s during ambulatory monitoring or for ≥10 s on a 12‐lead ECG); CBA‐related SAEs	Quality of life ([EQ‐5D], [AFEQT]), health care utilization
EARLY‐AF	Canada	Symptomatic AF for at least 24 months	Age <18 years, regular (daily) use of a Class 1 or 3 AAD, previous LA ablation or LA surgery, active intracardiac thrombus, LAD >5.5 cm, LVEF <35%, NYHA III‐IV, contraindication to OAC, eGFR <30 ml/min/1.73 m^2^, posterior wall thickness >1.8 cm	Use of a 28‐ or 23‐mm second‐generation cryoballoon (Arctic Front Advance, Medtronic)	Flecainide, propafenone, sotalol, dronedarone	The first recurrence of any AA (documented AF, AT, or AFL) lasting ≥30 s documented by continuous cardiac rhythm monitoring (Reveal LINQ, Medtronic).	The first recurrence of symptomatic AA (CCS‐SAF), AF burden, quality of life ([EQ‐5D], [AFEQT]), health care utilization, SAEs
Cryo‐FIRST	Europe, Australia, Latin America	Symptomatic PAF for at least 6 months	Age <18 years or >75 years, a history of successful or unsuccessful treatment of AF with a Class I or III AAD, previous LA ablation, LAD >4.6 cm, LVEF <50%, NYHA II‐IV, contraindication to OAC, interventricular septum thickness >1.2 cm, eGFR <60 ml/min	Use of a 28‐ or 23‐mm second‐generation cryoballoon (Arctic Front Advance Cardiac Cryoablation Catheter, Medtronic)	Flecainide, propafenone, sotalol, dronedarone	Freedom from any AA recurrence (at least one episode of AF, AT, or AFL) lasting >30 s documented by 7‐day Holter ECG or any other ECG recording	SAEs, recurrence of patient‐reported symptomatic palpitations

Abbreviations: AA, atrial arrhythmia; AAD, antiarrhythmic drug; AF, atrial fibrillation; AFL, atrial flutter; AT, atrial tachycardia; CBA, cryoballoon ablation; GFR, glomendar filtration rate; LA, left atrial: LAD, left atrial diameter; LVEF, left ventricular ejection fraction; OAC, oral anticoagulant; PAF, paroxysmal atrial fibrillation; SAEs, serious adverse events.

### 3.2 The primary outcomes

**FIGURE 2:**
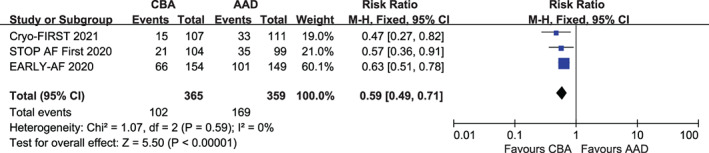
Forest plot illustrating the risk of recurrence of atrial arrhythmia during follow‐up among AF patients randomized to CBA versus AAD. AAD, antiarrhythmic drug; CBA, cryoballoon ablation

**FIGURE 3:**
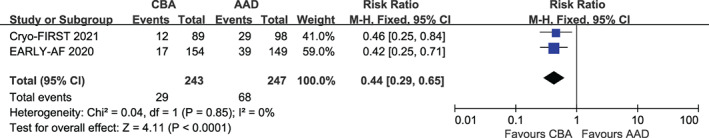
Forest plot illustrating the risk of recurrence of symptomatic atrial arrhythmias during follow‐up among AF patients randomized to CBA versus AAD. AAD, antiarrhythmic drug; CBA, cryoballoon ablation

Three trials comprising 724 patients reported recurrence of atrial arrhythmia during follow‐up. CBA was associated with a significant reduction in the recurrence of atrial arrhythmia compared with AAD, with low heterogeneity (RR, 0.59; 95% CI, 0.49–0.71; *p* < .00001; *I* ^*2*^ = 0%; Figure 2). Similarly, CBA was associated with less recurrence of symptomatic atrial arrhythmias compared with AAD during follow‐up (RR, 0.44; 95% CI, 0.29–0.65; *p* < .0001; *I* ^*2*^ = 0%; Figure 3).

### 3.3 The secondary outcomes

**FIGURE 4:**
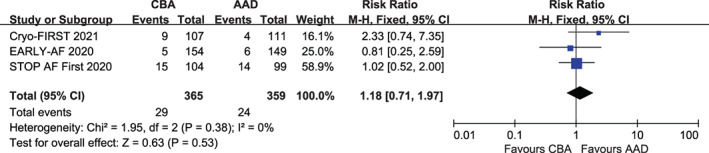
Forest plot illustrating the risk of SAEs during follow‐up among AF patients randomized to CBA versus AAD. AAD, antiarrhythmic drug; CBA, cryoballoon ablation; SAEs, serious adverse events

Table 3 summarizes the secondary outcomes and effect evaluations for patients with symptomatic AF who received CBA or AAD as initial therapy in the included trials. All three trials showed similar rates of SAEs in both groups (RR, 1.18; 95% CI, 0.71–1.97; *p* = .53; *I* ^2^ = 0%; Figure 4). The occurrence rate of additional ablation therapy (i.e., the proportion of patients who crossed over to the CBA therapy in the AAD group) was significantly higher after the failure of initial AAD therapy than after the failure of CBA as initial therapy (RR, 0.24; 95% CI, 0.06–0.89; *p* = .03; *I* ^*2*^ = 79%; Figure S1). A statistically non‐significant trend of more patients who crossed over to CBA group was also observed in the AAD group (RR, 0.14; 95% CI, 0.01–1.39; *p* = .09; *I* ^*2*^ = 90%; Figure S2). Moreover, there were no significant differences in other secondary outcomes (Table 3). The distribution of the rate of serious complications of CBA was as follows: TIA, one (0.3%) and transient phrenic nerve palsy, four (1.1%). There were no cases of death, stroke, cardiac tamponade, atrial esophageal fistula, or symptomatic PV stenosis at 12 months post‐CA. All four cases of phrenic nerve palsy resolved at the time of discharge. Table S1 provides detailed information on the serious complications associated with CBA.

**TABLE 3:** Secondary outcomes of AF patients who underwent CBA or AAD as initial therapy for symptomatic AF in the included trials

	No. of studies	Patients CBA/AAD	Events CBA/AAD	*p* value	Effect estimate RR (95% CI)	*I* ^2^ (%)
Bradycardia	2	258/248	2/3	.65	0.69 (0.14, 3.45)	0
Syncope	3	365/359	1/5	.17	0.33 (0.07, 1.60)	0
Chest pain	2	211/210	2/1	.62	1.65 (0.22, 12.25)	0
Pericardial complications	3	365/359	4/2	.48	1.65 (0.40, 6.79)	0
Vascular access site hemorrhage	2	261/260	2/0	.34	3.01 (0.31, 28.69)	0
Thromboembolic events	3	365/359	2/2	.99	0.99 (0.20, 4.80)	0

Abbreviations: AAD, antiarrhythmic drug; AF, atrial fibrillation; CBA, cryoballoon ablation; CI, confidence interval; RR, risk ratio.

### 3.4 Bias assessment and evidence classification

Table S2 provides a full summary of the risk of bias in the included trials. Due to the open label design, there is an existing risk of performance bias in the included RCTs. Using the GRADE system, all three study outcomes were considered high quality evidence (Table S3).

## 4 DISCUSSION

Most trials comparing CA with AAD in the treatment of AF patients with failure of initial pharmacotherapy showed that CA has an advantage over AAD therapy.^7^, ^8^, ^9^ Therefore, current guidelines and consensus statements recommend the use of CA for the management of drug‐refractory paroxysmal AF (Class I, Level A).^23^, ^24^ However, the recently published EAST‐AFNET 4 results revealed that in comparison to conventional treatment, early rhythm control by either ablation or medication led to better cardiovascular outcomes (including avoidance of stroke) at a median follow‐up of 5.1 years.^11^ Moreover, AF is a progressive disease.^25^ In the early stages of AF, it is triggered by one or more ectopic foci most commonly found in the pulmonary veins.^26^ Increasing evidence suggests that an earlier ablation intervention in the natural history of AF is more likely to prevent recurrence of atrial arrhythmia and halt disease progression by interrupting progressive pathophysiological changes.^26^, ^27^

In our analysis, we selected patients who were relatively younger and were early in the course of AF. The patients randomly assigned to receive CBA as initial therapy for symptomatic AF were significantly less likely to experience treatment failure within 12 months than those who received AAD. Our data are consistent with the results of observational studies involving patients who had not previously received treatment for rhythm control, in which a high percentage was found to have no recurrent atrial arrhythmia following CBA.^28^, ^29^ The results of this analysis support the current expert consensus recommending the use of CA as the first‐line treatment for paroxysmal AF, with a class IIa indication.^10^, ^30^ Three RCTs have previously compared RFA and AAD as first‐line treatment for symptomatic AF.^31^, ^32^, ^33^ In addition, a meta‐analysis by Hakalahti et al. involving 491 patients compared RFA with AAD as initial therapy, and showed a modestly lower risk of recurrent atrial arrhythmia with RFA than with AAD.^16^ However, these studies were limited by a small sample size^31^ or revealed only small differences in the recurrence rates of atrial arrhythmia.^33^ These results were also limited by a relatively high rate of re‐ablation and intermittent rhythm monitoring,^31^, ^32^, ^33^ thus negatively affecting the evaluation of differences between treatment groups. In the current investigation, there was a significantly lower rate of recurrent atrial arrhythmia with CBA than with AAD, and a lower incidence of AAD use and re‐ablation was observed in the CBA group. Furthermore, the EARLY‐AF study (Cryoablation or Drug Therapy for Initial Treatment of Atrial Fibrillation)^21^ used continuous rhythm monitoring, which is more sensitive than intermittent monitoring in the detection of paroxysmal AF.^34^ The FREEZE Cohort Study, which compared RFA and CBA as first‐line intervention for paroxysmal AF, has reported that the RFA group had more recurrences than the CBA group.^29^ However, further high‐quality head‐to‐head RCTs are required for the evaluation of the difference in efficacy between these frequently used ablation technologies as initial AF therapy.

Safety is an important consideration in the early use of ablation to treat AF. The iatrogenic complications from CA are generally more severe and immediate than the complications from drug therapy.^35^ Therefore, the risk is greater upfront. In our trial, the rate of SAEs was similar between the two groups. Serious complications of CBA were uncommon among patients who had not been previously treated for rhythm control. We observed no cases of death, stroke, cardiac tamponade, atrial esophageal fistula, or symptomatic PV stenosis after 12 months. However, the sample size may not be sufficiently large to detect uncommon outcomes. The most common periprocedural complication was transient phrenic nerve palsy, which is consistent with previous reports on the safety of CBA, possibly due to a large cooling surface area and deep lesions.^36^ However, the 1.1% incidence in our trial was still lower than the 2.7% and 13.5% reported in the FIRE AND ICE study (Cryoballoon or RFA for Paroxysmal AF)^37^ and the Sustained Treatment of Paroxysmal AF study (STOP AF),^9^ respectively. Overall, these results indicate that experienced operators can safely perform CBA procedures.

Although current guidelines recommend AAD treatment before CA, it has been shown that AAD treatment cannot prevent recurrence of AF in 43%–67% of patients,^38^ and it may be accompanied by extra‐cardiac adverse effects and severe pro‐arrhythmia.^10^ However, several previous adverse events related to AAD use were not observed in our study. Nevertheless, a relatively high proportion of patients randomly assigned in the AAD group crossed over to the CBA group within 12 months. These findings highlight the clinical challenges associated with AAD therapy for long‐term rhythm control.

The comparative outcomes between RFA and AAD cannot be extrapolated to studies on CBA versus AAD as first‐line therapy. Unlike in comparisons between CBA and AAD groups, there is no significant difference in the recurrence rate of symptomatic AF between RFA and AAD groups.^16^ Similarly, the MANTRA‐PAF trial (RFA as initial therapy in paroxysmal AF)^32^ reported that RFA failed to achieve the primary endpoint of reducing the AF burden, whereas there was a significant benefit in the EARLY‐AF study.^21^ In terms of safety, the distribution of SAEs in RFA were as follows: death (*n* = 1), stroke (*n* = 1), symptomatic PV stenosis (*n* = 1), and tamponade (*n* = 7).^16^ This was not observed in our study. In addition, compared with the prior meta‐analysis,^16^ the included trials were relatively homogeneous in methodology, with exception of arrhythmia monitoring. In the EARLY‐AF study,^21^ the insertion of an implantable cardiac monitor with daily transmissions was particularly sensitive in the detection of asymptomatic arrhythmia episodes.^34^ The EARLY‐AF trial accounts for approximately 60% of the weight in the current analysis; therefore, our results may be closer to the true recurrence rate than to those of the other two independent RCTs,^20^, ^22^ without underestimation.

It is worth noting that due to the potential extra‐cardiac toxicity of amiodarone, it was excluded from use in all included RCTs. However, amiodarone is the most effective drug for restoring and maintaining normal sinus rhythm.^39^ Directly avoiding the use of amiodarone may be problematic since the comparison was between the most advanced CBA and the least effective AAD. Low‐dose amiodarone might be considered as an initial therapy for AF, and trials comparing amiodarone with CBA are warranted in the future. To our knowledge, our study is the first meta‐analysis of CBA versus AAD as the initial therapy for symptomatic AF, showing the advantages of the former intervention over the latter. However, the included patients were relatively young and had mild to no cardiovascular disease, and the vast majority had paroxysmal AF. Hence, it remains unclear whether the results of our analysis can be applied to other patient groups or AF patients with severe structural heart disease. Further evaluation is necessary.

## 5 LIMITATIONS

Although this meta‐analysis only included RCTs, there were certain limitations. First, our analysis was based on study‐level data, not on data from individual participants, which could have greatly increased the validity of the comparison. Second, publication bias was not carried out due to the small number of qualified studies. Third, the included studies were methodologically inconsistent, particularly in the detection of arrhythmias during follow‐up. Using intermittent rather than continuous monitoring may have led to an overestimation of the treatment success rates in both groups. Fourth, the current study was not registered at the International Prospective Register of Systematic Reviews (PROSPERO), although we strictly followed the steps in conducting a systematic review. Finally, the follow‐up duration in the current trial was limited to 12 months, so we were unable to determine the long‐term impact of early ablation on AF progression. Further investigations are warranted.

## 6 CONCLUSION

Our meta‐analysis suggests that CBA is superior to AAD as a first‐line therapy for the prevention of recurrent atrial arrhythmia in relatively young patients with symptomatic paroxysmal AF and normal cardiac structure. Serious CBA‐related complications were observed to be uncommon. However, due to the open‐label design, the study limitations include small sample sizes, short follow‐up duration, and failure to reflect real‐world populations in the included RCTs. Thus, the results should be interpreted with caution. Larger patient populations and longer follow‐up periods are required to determine the beneficial role of early CBA in preventing degenerative processes that may lead to later recurrence of atrial arrhythmia.

## CONFLICT OF INTEREST

The authors report no conflicts of interest.

## Supporting information


**Data S1.** Supporting Information.

## Data Availability

All data generated or analyzed during this study are included in this article (and its Supporting information files).
