# Supplementary material for: Meta‐analysis of cryoballoon ablation versus antiarrhythmic drugs as initial therapy for symptomatic atrial fibrillation
Source: Clin Cardiol. 2021 Jul 29;44(10):1393–401. doi: 10.1002/clc.23695 (PMC8495081; doi:10.1002/clc.23695)
Supplement: Supplementary file 1 — Data S1. Supporting Information. [file CLC-44-1393-s001.docx]

**
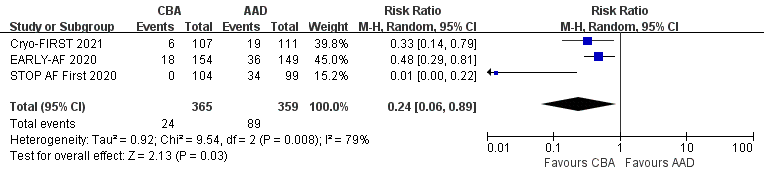
Figure S1** Forest plot illustrating the additional ablation after failure of initial treatment with either CBA or AAD. AAD: antiarrhythmic drug; CBA: cryoballoon ablation


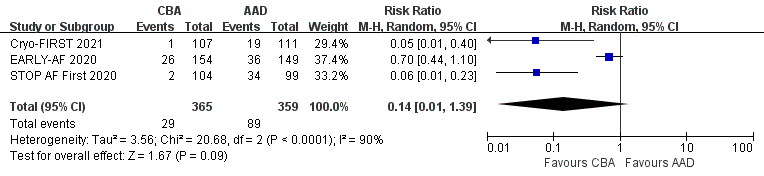


**Figure S2** Forest plot illustrating the percentage of cross-over to the alternative treatment

**Table S1** Serious complications related to CBA procedure

|  | **Death,**  **N (%)** | **Stroke/**  **TIA,**  **N (%)** | **Symptomatic PVS,**  **N (%)** | **Cardiac tamponade,**  **N (%)** | **AEF,**  **N (%)** | **Transient PNP,**  **N (%)** |
| --- | --- | --- | --- | --- | --- | --- |
| STOP AF First (N=104) | 0 (0.0%) | 0 (0.0%) | 0 (0.0%) | 0 (0.0%) | 0 (0.0%) | 0 (0.0%) |
| EARLY-AF (N=154) | 0 (0.0%) | 0 (0.0%) | 0 (0.0%) | 0 (0.0%) | 0 (0.0%) | 3 (1.9%) |
| Cryo-FIRST (N=107) | 0 (0.0%) | 1 (0.9%) | 0 (0.0%) | 0 (0.0%) | 0 (0.0%) | 1 (0.9%) |
| **CBA (N=365)** | **0 (0.0%)** | **1 (0.3%)** | **0 (0.0%)** | **0 (0.0%)** | 0 (0.0%) | **4 (1.1%)** |

AEF: atrial esophageal fistula; CBA: cryoballoon ablation; RFA: radiofrequency ablation; PNP: phrenic nerve palsy; PVS: pulmonary vein stenosis; TIA: transient ischemic event

**Table S2** Risks of bias in the included trials according to the Cochrane risk of bias assessment tool

| **Study/(year)** | **Random**  **sequence**  **generation**  **(selection bias)** | **Allocation**  **concealment**  **(selection bias)** | **Blinding**  **of participants**  **and personnel**  **(performance**  **bias)** | **Blinding**  **of outcome**  **assessment**  **(detection**  **bias)** | **Incomplete**  **outcome**  **data**  **(attrition**  **bias)** | **Selective**  **reporting**  **(reporting**  **bias)** | **Other bias** |
| --- | --- | --- | --- | --- | --- | --- | --- |
| STOP AF First (2020) | Low | Unclear | High | Low | Low | Low | Low |
| EARLY-AF (2020) | Low | Unclear | High | Low | Low | Low | Low |
| Cryo-FIRST (2021) | Low | Unclear | High | Low | Low | Low | Low |

**Table S3** The quality of evidence of each outcome of interest based on GRADE assessment tool

| **Number of study (Study design)** | **Limitations**  -No serious  -Serious  -Very serious | **Inconsistency**  -No serious  -Serious  -Very serious | **Indirectness**  -No serious  -Serious  -Very serious | **Imprecision**  -No serious  -Serious  -Very serious | **Publication bias**  -Detected  -Undetected | **Quality of evidence**  -High  -Moderate  -Low  -Very low |
| --- | --- | --- | --- | --- | --- | --- |
| **Atrial arrhythmia recurrence** | | | | | | |
| 3 RCTs | No serious | No serious | No serious | No serious | Undetected | High |
| **Symptomatic atrial arrhythmias recurrence** | | | | | | |
| 2 RCTs | No serious | No serious | No serious | No serious | Undetected | High |
| **SAEs** | | | | | | |
| 3 RCTs | No serious | No serious | No serious | No serious | Undetected | High |

RCTs: randomized controlled trials; SAEs: serious adverse events
